# Proposal of a Machine Learning Based Prognostic Score for Ruptured Microsurgically Treated Anterior Communicating Artery Aneurysms

**DOI:** 10.3390/jcm14020578

**Published:** 2025-01-17

**Authors:** Massimiliano Minardi, Andrea Bianconi, Luca Mesin, Luca Francesco Salvati, Federico Griva, Alessandro Narducci

**Affiliations:** 1 Neurosurgery, Santa Croce and Carle Hospital, 12100 Cuneo, Italy; 2 Neurosurgery, IRCCS Policlinico S. Martino, 16132 Genova, Italy; 3 Department of Neuroscience, Rehabilitation, Ophthalmology, Genetics and Maternal and Child Health (DINOGMI), University of Genoa, 16126 Genova, Italy; 4 Department of Electronics and Telecommunications, Polytechnic University of Turin, 10129 Turin, Italy; luca.mesin@polito.it; 5 Neurosurgery, Santa Corona Hospital, 17027 Pietra Ligure, Italy; 6 Neurosurgery, San Giovanni Bosco Hospital, 10154 Turin, Italy

**Keywords:** AcoA aneurysm, machine learning, clipping, SAH

## Abstract

**Background:** Aneurysmal subarachnoid hemorrhage (aSAH) carries significant mortality and disability rates, with rebleeding posing a grave risk, particularly in anterior communicating artery (AcoA) aneurysms. This retrospective study aims to analyze preoperative and intraoperative variables of patients with ruptured AcoA aneurysms, evaluating the association of these variables with patient outcomes using machine learning techniques, proposing a prognostic score. **Materials and Methods**: A retrospective study was conducted on 50 patients who underwent microsurgical clipping for a ruptured AcoA aneurysm at San Giovanni Bosco Hospital, Turin, Italy. The clinical and aneurysmal data—including clinical evaluations, risk factors, aneurysmal characteristics, and intra- and postoperative details—were examined. The study population was analyzed using machine learning techniques such as the MRMR algorithm for feature selection, and the LASSO method was employed to construct linear predictive models based on these features. **Results:** The study cohort had a mean age of 54 years, with 26 female and 24 male patients. Temporary clipping of main vessels was performed in 96% of procedures, with a mean duration of 3.74 min. Postoperatively, the mean Intensive Care Unit (ICU) stay was 7.28 days, with 14% mortality at 30 days and 4% within the first week. At the six-month follow-up, 63% of discharged patients had a Glasgow outcome scale (GOS) of 5, with radiological confirmation of complete aneurysm exclusion in 98% of cases. Machine learning techniques identified the significant predictors of patient outcomes, with LASSO algorithms generating linear models to predict the GOS at discharge and at 6 months follow-up. **Conclusions:** Preoperative factors like the BNI score, Vasograde, and preoperative cerebral edema demonstrate significant correlations with patient outcomes post-clipping. Notably, intraoperative bleeding and extended temporary clipping durations (over 3 min) emerge as pivotal intraoperative considerations. Moreover, the AcoA prognostic score shows promise in predicting patient outcomes, discharge plans, and ICU duration.

## 1. Introduction

According to estimates, the overall worldwide incidence of aneurysmal subarachnoid hemorrhage (aSAH) is approximately 6.1 cases per 100,000 person-years [1]. The incidence rates tend to be higher in the Finnish and Japanese populations, with incidences of around 16.6 and 28 per 100,000 person years, respectively [1].

The degree of disability and mortality after aSAH depends on many variables: possible rebleeding, associated comorbidities, Hunt-Hess and modified Fisher grade at onset, occurrence of vasospasm, etc. Among all factors, rebleeding is the most ominous event that can happen to the patient. In fact, roughly 70–90% of cases that rebleed result in death [2]. The risk of a second bleeding is highest during the first days; therefore, the timing of treatment is paramount: early aneurysm occlusion can significantly reduce in-hospital mortality of aSAH [2,3].

About 30% of cerebral aneurysms are located in the anterior communicating artery (AcoA) complex [4]. Some studies have shown that AcoA aneurysms have a greater tendency to rupture, even in the case of small lesions [5]. Ruptured AcoA aneurysms must be treated as soon as possible from the first bleeding: endovascular (coiling) and microsurgical (clipping) options represent two possible ways to close the vascular malformation, comparable in terms of clinical outcome in a long period of follow-up.

The aim of this study is to analyze the preoperative and intraoperative factors that influence the outcome of microsurgical treatment of ruptured AcoA aneurysms, finally proposing a machine-learning-based prognostic score.

## 2. Materials and Methods

### 2.1. Study Population

We conducted a retrospective single-center cohort study including 50 patients who underwent microsurgical clipping for a ruptured AcoA aneurysm at San Giovanni Bosco Hospital (Turin) between April 2014 and April 2021. The follow-ups were prolonged until January 2022. The exclusion criteria included aneurysms located outside the AcoA complex, unruptured aneurysms, and patients who received endovascular treatment as the first treatment. Patients aged < 18 years were excluded. All patients who could not undergo a minimum follow-up of six months were excluded. The microsurgical procedures were performed by three experienced neurovascular surgeons.

The Glasgow outcome scales at discharge and at 6 months after discharge were considered primary outcomes.

### 2.2. Clinical and Aneurysmal Features

On admission, patients underwent computerized tomography (CT) scan and CT-Angiogram (CTA) when SAH was found. The clinical evaluation scales used during the first assessment were as follows: the World Federation Neurosurgical Society grading score (WFNS score) and the Hunt-Hess score (H-H score). All radiological data were reviewed using the modified Fisher scale and BNI score [6]. Vasograde was then calculated in all patients [7,8].

The risk factors and the use of anticoagulant or anti-platelet therapies were investigated throughout the cohort of patients. The presence of preoperative cerebral edema was evaluated according to the Subarachnoid Hemorrhage Early Brain Edema Score (SEBES) classification (for simplification, we assigned the score 3–4 as positive for the presence of edema) [9].

The aspect ratio, dome–neck ratio, and size ratio were calculated for all aneurysms. Furthermore, the presence or absence of a bleb, the orientation of the dome, the rotation of the AcoA complex, and the presence of anatomical variants were also evaluated. Several factors were examined during the surgical phase, including the length of procedure, type and side of the surgical approach, the number of aneurysms treated, whether an external ventricular drain (EVD) was positioned or not, the use and length of temporary clipping, and whether the lamina terminalis was opened.

In the postoperative period, variables examined were the presence of acute postoperative cerebral edema, presence of vasospasm and its treatment, occurrence of delayed cerebral ischemia (DCI) related to vasospasm, onset of convulsive seizures, development of chronic hydrocephalus, the length of stay in the Intensive Care Unit (ICU), the type of discharge disposition, and aneurysm retreatment. The Glasgow outcome scale (GOS) was calculated at discharge and at 6–12 months follow-up.

### 2.3. Machine Learning and Statistical Analysis

The features described above were first explored by basic descriptive statistical tools (e.g., box plots, correlation) to investigate the possible relationships with the following outcomes of interest: GOS at discharge and at 6 months follow-up.

Overall, the following list of preoperative features was obtained: risk factor, Vasograde, dome orientation, Fisher grade, BNI score, brain swelling, hydrocephalus, hypertension, time between presentation and surgery, aneurysm width, aneurysm Dmax, aneurysm height, aneurysm neck size, dome–neck ratio, aspect ratio, size ratio, dome rotation, and blebs. In addition, the following intra- and postoperative features were included: length of surgery (in minutes), intraoperative EVD, lamina terminalis opening, temporary clipping, duration of temporary clipping (in minutes), intraoperative bleeding, immediate postoperative brain swelling, vasospasm, DCI, and length of ICU stay (in days).

The minimum redundancy maximum relevance (MRMR) algorithm was used to determine which features predicted the outcomes [10]. The MRMR method searches for the subset of features that share the maximum mutual information with the desired outcome but have a minimum redundancy (in terms of mutual information among the features of the set). The six features with the highest scores (computed by the MRMR) in predicting each of the two outcomes of interest (GOS at discharge and 6 months follow-up) were selected. Then, information from these selected input features was integrated to estimate the outcomes of interest. The least absolute shrinkage and selection operator (LASSO) was used to estimate a linear combination of features that reduces the residual error with respect to the outcomes [11].

## 3. Results

### 3.1. Characteristics of Patients and Aneurysms

Mean age of the cohort was 54 years (±13). Twenty-six patients were female, and 24 were male. Table 1 and Table 2 lists the number of cases for each preoperative examined factor and the preoperative morphological characteristics.

In all patients, standard pterional approach was performed: 24 (48%) patients with left access and 26 (52%) with right approach. The mean surgical time was 211.16 min (SD 57.83). In 39 (78%) patients, an EVD was positioned to facilitate the surgical maneuvers. The lamina terminalis was opened in 16 (32%) patients. In 48 (96%) procedures, temporary clipping of at least 2 of the 5 main vessels was performed, with a mean clipping time of 3.74 min (SD 2.44).

Of 50 patients, 13 (26%) experienced intraoperative bleeding, and 6 (12%) received a gyrus rectus resection to better expose the AcoA complex (Table 3).

Table 4 summarizes the postoperative characteristics. The mean ICU length of stay was 7.28 days (SD 8.21). Mortality at 30 days was 14%, while 4% died in the first week. 46% of patients were discharged: while 83% had a GOS of 5, only 17% had a GOS of 4. Rehabilitation treatment was required in 40% of cases. Of these, 35% were transferred to the facility with a GOS of 4, 60% with a GOS of 3, and 5% with a GOS of 2. The minimum follow-up was 6 months (7–60 months). After this period, 63% of discharged patients had a GOS of 5, 19% a GOS of 4, 16% a GOS of 3, and the remaining 2% a GOS of 2. For the remaining patients, the GOS measurements remained stable.

At 6 months follow-up, 98% of patients demonstrated radiological confirmation of complete exclusion of the aneurysm. In 2% of cases, there was minimal residual, which was treated endovascularly 12 months afterward with complete exclusion.

### 3.2. Machine Learning and Clinical Prognostic Score

**Figure 1:**
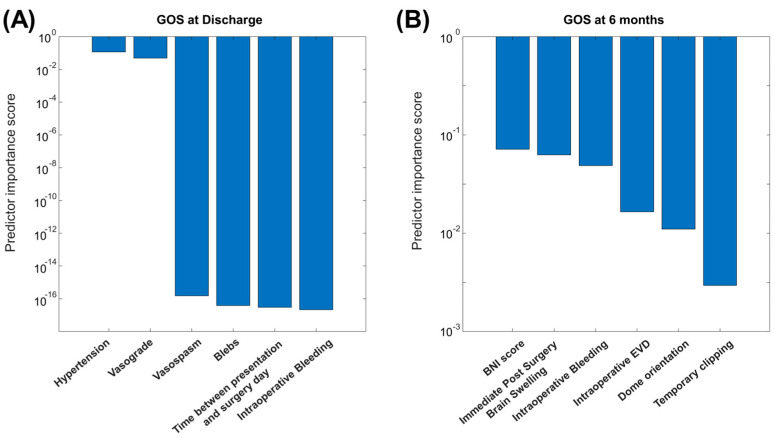
Best features indicated by the minimum redundancy maximum relevance (MRMR) algorithm. (**A**) MRMR scores of the best 6 features for predicting the GOS at discharge (the scores of the features number 4 to 6 are very small, in the order of 10–15, but still much larger than those of subsequent features, which decrease by orders of magnitude after the first 6). (**B**) MRMR scores of the best 6 features for predicting the GOS at 6 months.

Figure 1 shows the minimum redundancy maximum relevance (MRMR) scores of the best 6 predictors for each of the outcomes of interest.

**Figure 2:**
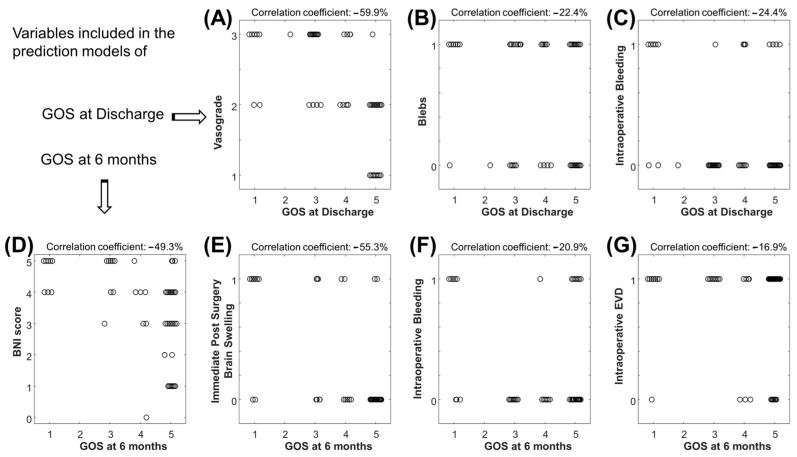
Scatter plot of features selected by the least absolute shrinkage and selection operator (LASSO) to predict the GOS at discharge or at 6 months follow-up. (**A**) Vasograde versus GOS at discharge. (**B**) Blebs versus GOS at discharge. (**C**) Intraoperative bleeding versus GOS at discharge. (**D**) BNI score versus GOS at 6 months follow-up. (**E**) Immediate post-surgery brain swelling versus GOS at 6 months follow-up. (**F**) Intraoperative bleeding versus GOS at 6 months follow-up. (**G**) Intraoperative EVD.

The best 6 features indicated by the MRMR algorithm were then processed by the LASSO algorithm, which makes a further selection of the best features for a linear model approximating the output of interest. Figure 2 shows the scatter plots of the features selected by LASSO and used to predict either the GOS at discharge or at 6 months follow-up.

**Figure 3:**
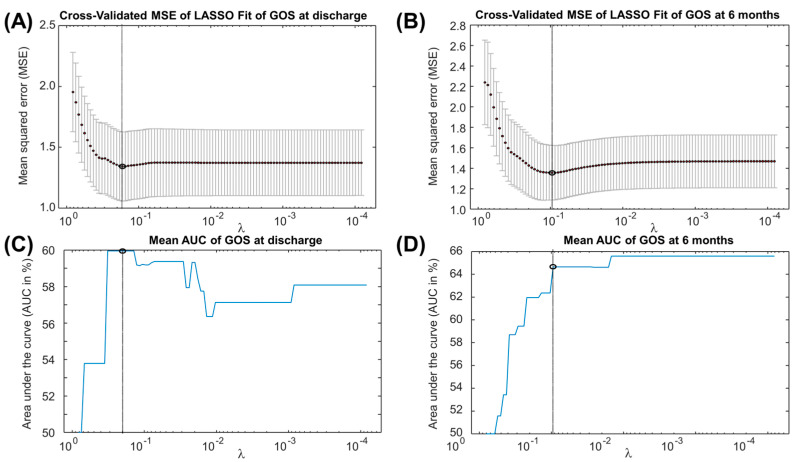
Mean squared error (MSE) and area under the curve (AUC) of the linear model selected by LASSO as a function of the regularization coefficient λ. (**A**) Mean MSE and error bars in cross-validation test of the LASSO fit of GOS at discharge, with indication of the selected coefficient λ. (**B**) Same as (**A**), but for the fit of the GOS at 6 months follow-up. (**C**) AUC of the LASSO fit of GOS at discharge, with indication of the optimal λ. (**D**) Same as (**C**), but for the fit of the GOS at 6 months follow-up.

Figure 3 shows the cross-validation error and the area under the curve (AUC) of the linear models estimated by LASSO as a function of the regularization coefficient. Specifically, the linear estimation model obtained by LASSO to predict the GOS at discharge is the following (rounded weights with 2-digit precision):

GOS at discharge = 5.90 − 1.06 × Vasograde − 0.64 × Blebs − 0.51 × Intraoperative Bleeding(1)

The formula provided by LASSO to estimate GOS at 6 months follow-up is the following:

GOS at 6 months follow-up = 5.61 − 0.37 × BNI Score − 1.54 × Immediate Postoperative Brain Swelling − 0.75 × Intraoperative Bleeding − 0.29 × Intraoperative EVD(2)

**Figure 4:**
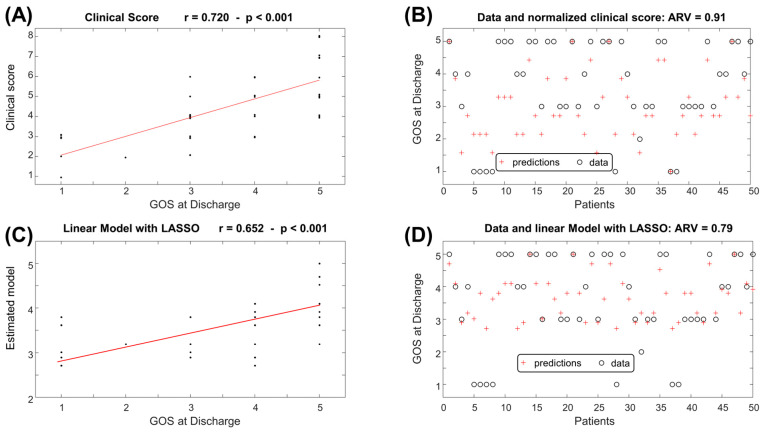
Relationship between clinical score or linear estimation model (given in Equation (1)) and GOS at discharge. (**A**) Scatter plot of the clinical score versus the GOS at discharge. The interpolation line is also shown in red (r: correlation coefficient) (**B**) GOS at discharge compared to the clinical score, normalized by applying a linear map to have a range between 1 and 5 (see Equation (3)). (**C**) Scatter plot of the linear estimator provided by LASSO versus the GOS at discharge. (**D**) GOS at discharge compared to the linear estimation model. ARV: average rectified value of the estimation error.

**Figure 5:**
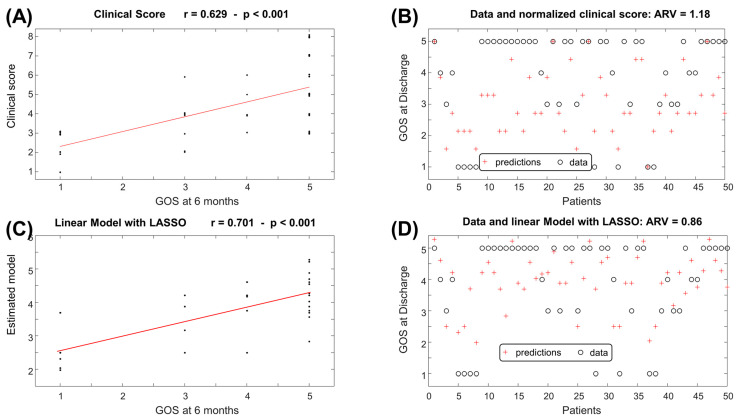
Relationship between clinical score or linear estimation model (Equation (2)) and GOS at 6 months follow-up. (**A**) Scatter plot of the clinical score versus the GOS at 6 months follow-up. (**B**) GOS at 6 months follow-up compared to the clinical score, normalized by applying a linear map to have a range between 1 and 5 (Equation (3)). (**C**) Scatter plot of the linear estimator provided by LASSO versus the GOS at 6 months follow-up. (**D**) GOS at 6 months follow-up compared to the linear estimation model.

Figure 4 and Figure 5 show the prediction of the GOS at discharge and at 6 months follow-up, respectively, with a comparison to the clinical prognostic score. Note that the outputs of the clinical score were normalized to get a range between 1 and 5, using the following scaling:

Normalized Clinical Score = 5 × (Clinical Score − 1)/7 + 1(3)

**Figure 6:**
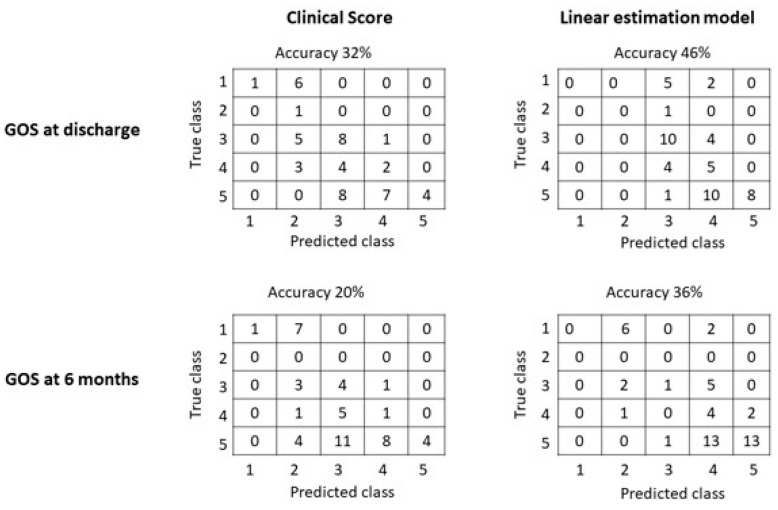
Confusion matrices of the estimation of GOS at discharge or at 6 months follow-up, using either the normalized clinical score (Equation (3)) or the linear estimation models (provided by Equations (1) and (2) for the GOS at discharge and at 6 months follow-up, respectively). Tolerating a maximum error of 1 class (thus keeping correct the prediction if the actual GOS is at maximum 1 class apart), the following accuracies are obtained: 78% and 84% for the GOS at discharge using the clinical score and LASSO, respectively; 68% and 92% for the GOS at 6 months follow-up using the clinical score and LASSO, respectively.

Taking the approximation to the closest integer of the normalized clinical score and of the outputs of the linear estimation models (1) and (2), the outcomes of interest were estimated. Figure 6 shows the corresponding confusion matrices. Notice that most of the errors were among close classes. For example, maintaining the predictions when the true value was at maximum 1 class apart, the accuracy of the clinical score was 78% and 68% for the GOS at discharge and at 6 months follow-up, respectively; when using the linear model obtained by LASSO, the accuracy was 84% and 92% for the GOS at discharge and at 6 months follow-up, respectively.

### 3.3. Prognostic Score

**Figure 7:**
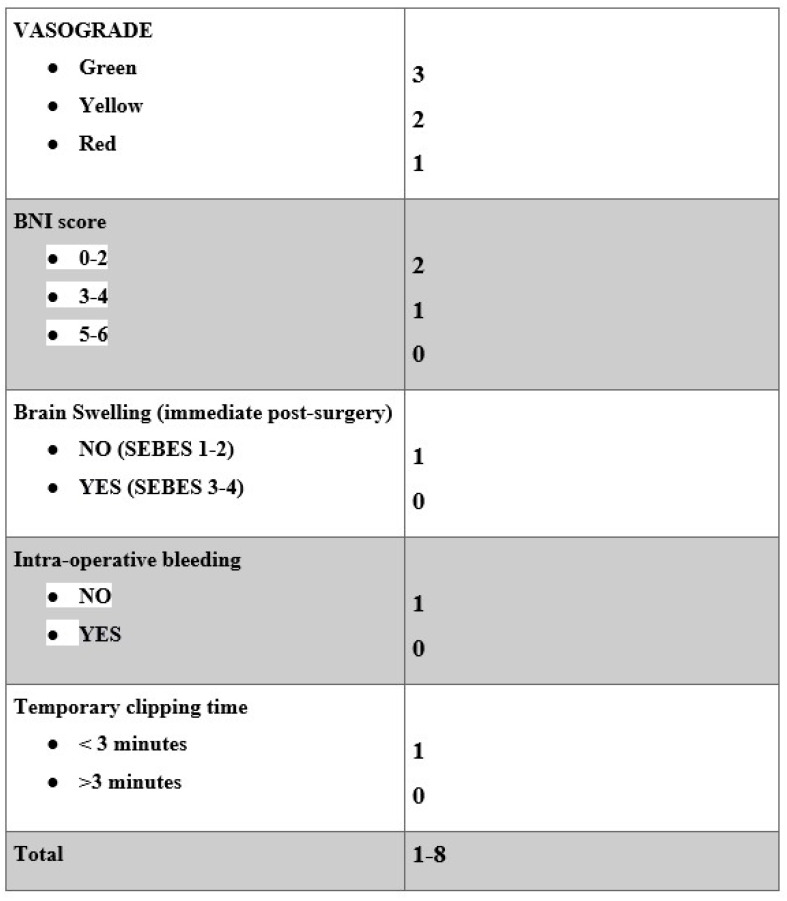
Final machine-learning-based prognostic score.

The features selected by our machine learning approach represent the cradle of the most significant preoperative and intraoperative parameters. The outcome correlations are shown in Figure 2. Although the models have poor accuracy in identifying low values of GOS, they demonstrate good average predictions of the outcomes of interest (Figure 3, Figure 4 and Figure 5). Additionally, the models reflect the unbalanced representation of the different classes in our dataset, including only 8 patients with a GOS of either 1 or 2. If an error of maximum 1 score is tolerated in the prediction, the proposed models show accuracies of 84% for the GOS at discharge and 92% for the GOS at 6 months follow-up. They were used to build an AcoA prognostic score that represents an easy and convenient attempt to describe the severity of patients with SAH (Figure 7).

## 4. Discussion

Anterior communicating artery (AcoA) aneurysms are among the most common aneurysms in anterior circulation and are particularly prone to rupture [12]. With the development of new endovascular techniques, treatment options continue to be debated [13]. Several studies have shown that both microsurgical clipping and endovascular coiling yield comparable long-term outcomes [14,15,16,17]. According to the Barrow Ruptured Aneurysm Trial (BRAT), no significant difference was found in long-term outcomes between the two treatment groups [17]. The coiling option in the International Subarachnoid Aneurysm Trial (ISAT) is associated with a lower intraoperative risk compared to clipping, yet both methods show similar long-term results [18]. Retrospective and comparative studies, such as the Cerebral Aneurysm Rerupture After Treatment (CARAT), have demonstrated that the percentage of complete aneurysm occlusion is a key predictive factor for the risk of recurrence and rebleeding. The success rate for achieving complete and long-term occlusion is lower for coiling compared to other methods [19,20].

The growing prominence of machine learning in neurosurgery underscores its increasing relevance in guiding clinical decisions [21,22]. Some studies used machine learning to predict aneurysm rupture [23] and the outcome of treatment [24]. Here, we applied simple machine learning approaches to our dataset in order to provide linear models for the prediction of the GOS at discharge and after 6 months. Many features, including pre-, intra-, and post-surgery, have been examined. Their relevance has been investigated by the MRMR, which yielded a relevance score by removing redundancy and considering the non-linear relationships with the outcomes of interest (Figure 1). Then, LASSO was applied to the best features selected by MRMR to make a further selection of the essential features and to give a linear model for the prediction of the outcome.

The integration of clinical experience and data-driven processing has led us to the development of simple prognostic scores that, if supported by future studies on extended datasets, could provide useful insights.

### 4.1. Preoperative and Intraoperative Parameters

Given the ongoing debate between surgical and endovascular techniques, much of the scientific literature has focused on comparing the outcomes of coiling and clipping, with less emphasis on the in-depth evaluation of preoperative variables that may influence outcomes in patients undergoing clipping [25,26]. In the context of ruptured AcoA aneurysms treated with clipping, addressing this knowledge gap becomes crucial. Several key factors should be considered. The microsurgical treatment of AcoA aneurysms can be significantly influenced by the precise location of the aneurysm relative to the surrounding neurovascular structures, which plays a critical role and may potentially predict outcomes [27,28]. The development of symptomatic infarction requiring surgical removal is a notable predictor of functional outcomes. Additionally, the complexity of local angioarchitecture makes the anterior circulation particularly vulnerable to ischemic damage [29,30]. It is also important to note that the negative effects of temporary vessel occlusion on cognitive function, occurring before ischemic damage, are significant. These cognitive changes should not be underestimated during surgical procedures involving temporary clipping [31]. In our study, both statistical and machine learning analyses demonstrated that preoperative clinical factors such as Vasograde, BNI score, and preoperative cerebral edema are strongly negatively correlated with the Glasgow outcome scale (GOS) at discharge and at the 6-month follow-up. This finding highlights the prognostic value of these indices. Specifically, Vasograde and comorbidities appear to play a decisive role in the development of chronic hydrocephalus and long-term seizures. Two main factors contribute to this trend: the increased fragility of the patient due to comorbidities, and the larger extent of bleeding, which raises the risk of convulsive seizures and hydrocephalus due to altered cerebrospinal fluid dynamics.

### 4.2. Prognostic Score

Considering specifically the proposed prognostic score, Vasograde is the most significant determinant of clinical prognostic score, and it has the strongest association with clinical outcome. Machine learning helped to test this clinical score in determining these results. The GOS at discharge tends to be higher when the prognostic score increases (*p*-value < 0.001). This score also shows a good correlation with the 6 months follow-up. Based on the data analyzed by machine learning, it is possible to assume that patients with massive and marked neurological deterioration at onset (low Vasograde score) have a lower tolerance to temporary clipping. When the brain is suffering from extensive SAH, even a few minutes of cerebral blood flow variation in some districts could worsen long-term general conditions. In summary, our analysis underscores the pivotal role of preoperative variables, including Vasograde, BNI score, and preexisting cerebral edema, in predicting outcomes for patients with AcoA aneurysms. By emphasizing Vasograde and other impactful parameters, our score demonstrates a robust ability to predict favorable outcomes, both at discharge and at the 6-month follow-up, showcasing its utility in assessing long-term prognosis.

### 4.3. Study Limitations

A relatively small sample size and infrequent complications, associated with bias selection, may affect the statistical power of this retrospective study. Future expansion of the dataset could lead to the development of more precise and stable interpretations, and maybe other features are statistically important. A prospective study should be conducted to enhance the statistical validity, particularly in relation to other scores. Probably this type of score could be extended to all anterior circulation aneurysms. It would be valuable to conduct an analysis and compare the outcomes with those of endovascularly treated cases, possibly developing a comprehensive and standardized score applicable to all treatment options.

## 5. Conclusions

Our results highlight the feasibility of surgical clipping in achieving long-term complete aneurysm occlusion. Preoperative factors such as the BNI score, Vasograde, and preoperative cerebral edema (SEBES) exhibit a strong correlation with the outcome of patients treated with clipping. Intraoperative bleeding and prolonged temporary clipping times (above 3 min) emerge as the most important intraoperative variables. The AcoA prognostic score seems to be able to predict patient outcomes, discharge disposition, and duration of stay in the ICU.

## Figures and Tables

**Table 1:** Aneurysm preoperative examined factor.

Pre-Op Features	N. Cases and %
Hunt-Hess:	
I–II	29 (58%)
III–IV	18 (36%)
V	3 (6%)
WNFS:	
I–II	32 (64%)
III–IV	14 (28%)
V	4 (8%)
Modified Fisher:	
I–II	13 (26%)
III	14 (28%)
IV	23 (46%)
Vasograde:	
Green	8 (16%)
Yellow	21 (42%)
Red	21 (42%)
BNI score:	
0–I–II	10 (20%)
III–IV	26 (52%)
V	14 (28%)
Brain swelling	26 (52%)
Pre-op hydrocephalus	23 (46%)
Risk factors:	
<or equal to 2 comorbidities	40 (80%)
3–4 comorbidities	9 (18%)
>4 comorbidities	1 (2%)

**Table 2:** Aneurysm morphological features.

Morphological Features	Mean	Standard Deviation	N. Cases and %
Neck An. (mm)	3.56	1.05	
High An. (mm)	6.33	2.64	
Dmax An. (mm)	7.5	2.88	
Width An. (mm)	5.27	2.55	
Aspect ratio	1.79	0.67	
Dome–neck	1.89	0.72	
Size ratio	3.71	1.98	
Blebs			29 (58%)
Complex rotation			5 (10%)
Dome orientation:			
Superior			22 (44%)
Inferior			17 (34%)
Anterior			25 (50%)
Posterior			6 (12%)
Asymmetry A1			23 (46%)
Heubner’s Duplication			2 (4%)

**Table 3:** Aneurysm intraoperative features.

Intraoperative Features	Mean	Standard Deviation	N. Cases (%)
Side approach			Dx 26 (52%) Sin 24 (48%)
Surgical time	211.16 min	57.83	
DVE			39 (78%)
Opening lamina terminalis			16 (32%)
Parenchymal resection			6 (12%)
Temporary Clipping	3.74 min	2.44	48 (96%)
Intraoperative bleeding			13 (26%)
Brain swelling			8 (16%)

**Table 4:** Aneurysm postoperative features.

Postoperative Features	N. Cases (%)
Post-op. bleeding	3 (6%)
Post-op. ischemia	13 (26%)
Post-op. brain swelling	13 (26%)
Vasospasm	24 (48%)
Vasospasm treatment:	13 (26%)
Oral nimodipine	12 (92.3% of cases treated)
DSA + intraarterial nimodipine	4 (30.77% of cases treated)
VP shunt	12 (24%)
Seizures	6 (12%)
Death	7 (14%)
GOS at discharge:	
2	1 (2.33%)
3	12 (27.91%)
4	11 (25.58%)
5	19 (44.19%)
GOS at 6 months:	
2	1 (2.33%)
3	7 (16.27%)
4	8 (18.6%)
5	27 (62.8%)
Aneurysm residual	1 (2.32%)
Retreatment at 6–12 months	1 (2.32%)

## Data Availability

The datasets that were generated and analyzed during the current study are available upon request from the corresponding author in a reasonable manner.
